# Natural rubber – Increasing diversity of an irreplaceable renewable resource

**DOI:** 10.1016/j.heliyon.2024.e25123

**Published:** 2024-01-29

**Authors:** Judit E. Puskas, Katrina Cornish, Boguspaev Kenzhe-Karim, Meirambek Mutalkhanov, Gabor Kaszas, Kristof Molnar

**Affiliations:** a Department of Food, Agricultural and Biological Engineering, College of Food, Agricultural and Environmental Science, The Ohio State University, 1680 Madison Avenue, Wooster, OH 44691, USA; b Department of Horticulture and Crop Science, College of Food, Agricultural and Environmental Science, The Ohio State University, 1680 Madison Avenue, Wooster, OH 44691, USA; c Al-Farabi Kazakh National University, Department of Biotechnology, 71 al-Farabi Ave., Almaty, Kazakhstan; d Laboratory of Nanochemistry, Department of Biophysics and Radiation Biology, Semmelweis University, Nagyvarad ter 4. Budapest, 1089, Hungary

**Keywords:** Natural rubber, *Scorzonera tau-saghyz*, Extraction, High-resolution size exclusion chromatography, Potential U.S. domestic rubber supply

## Abstract

This paper discusses the importance of introducing domestic natural rubber production and presents the rediscovery of a rubber-producing species, *Scorzonera tau-saghyz* or “mountain gum”, originally discovered in 1929 on the Karatau mountains in Kazakhstan. This plant could potentially also be cultivated in the U.S. In this exploratory work, roots (2–5 years old) were harvested on June 16, 2021 from wild strands in the Karatau mountains, Kumantas ridge, and Saraba, Kazakhstan, and processed at the Ohio State University. The rubber extraction method was based on an indigenous method in Kazakhstan to make natural chewing gum. Water extraction followed by purification yielded 16.2 wt% rubber from the dry roots, in comparison with 4–8 wt% from most rubber dandelion (*Taraxacum kok-saghyz)* plants, also a potential domestic rubber producing plant. High-resolution size exclusion chromatography was used to analyze rubber samples. The molecular weights and gel and oligomer contents were very similar to the rubber from *Hevea brasiliensis*, the current commercial source of natural rubber. More detailed investigations of this very interesting rubber-producing plant are in progress.

## Graphical abstract

**Figure:**
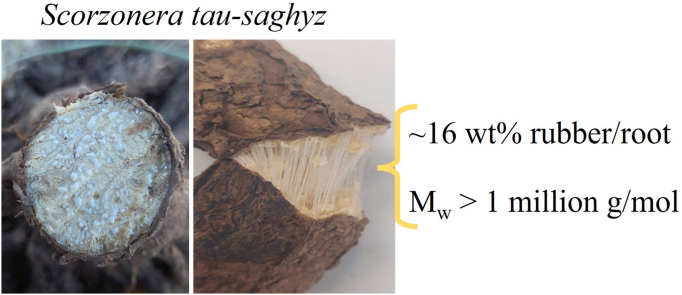

## 1 Introduction

### 1.1 The need for NR supply security

**Fig. 1:**
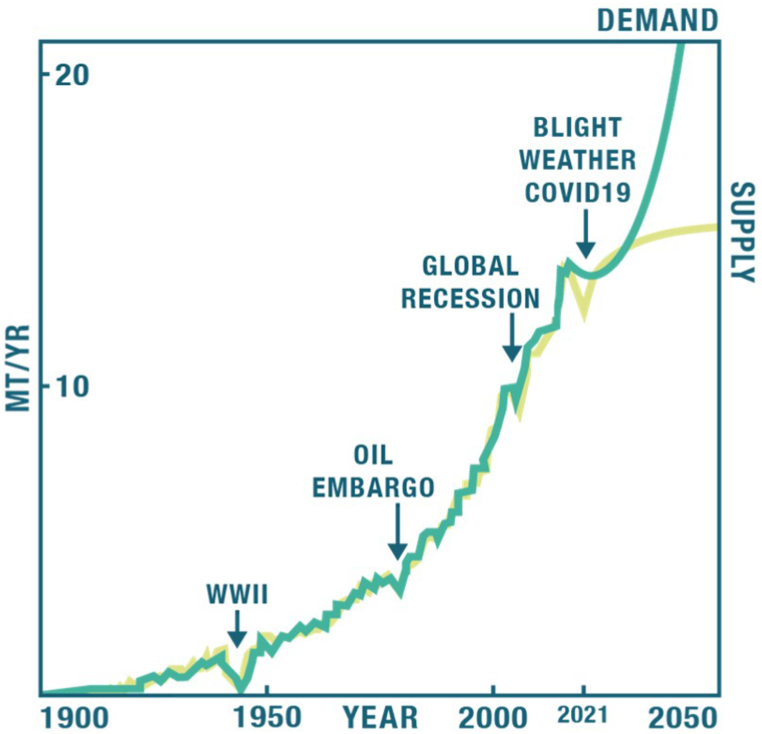
Global supply and demand showing perturbations caused by crises. WWII: World War II. MT/YR: Million tons per year.

A 2015 Rubber Journal Asia article asked, “What would industrial progress be without natural rubber (NR)? It's hardly imaginable” [1]. NR has a unique combination of properties, including abrasion resistance, unique dynamic mechanical properties, flexibility and strength, and is irreplaceable by any synthetic rubber (SR) in many products (e.g., car and airplane tires, vibration isolators, specialty medical gloves) [2]. The Critical Agricultural Materials Act of 1942 (last amended in 2018) [3,4] states that "*natural latex rubber is a commodity of vital importance to the economy, defense and general well-being of the Nation*." The para, or Brazilian rubber tree (*Hevea brasiliensis*), the single commercial source globally, can grow only in tropical climates, and the U.S. imports 100 % of its NR supply as well as many finished NR-containing goods. Many tropical plants produce rubber [5] but most either cannot be grown as crops or their extracted rubber is not of sufficient quality. In general, demand for, and supply of, NR have been growing exponentially (Fig. 1) [6,7] but is perturbed by major world events. The global supply sharply dropped in 2020 due to extreme weather, labor shortages exacerbated by COVID-19 and fungal blights which spread to more than 500,000 ha across SE Asia, destroying entire Hevea plantations [8]. Hevea rubber production cannot meet projected U.S. and global market demands (Fig. 1) partly due to the global moratorium on clear-cutting rain forests to plant more rubber trees. With no domestic NR production, total dependence on foreign sources threatens U.S. rubber security and, recently, U.S. manufacturers experienced significant supply disruptions [9]. The doubling of demand for protective gloves brought about by the COVID-19 pandemic required an additional million tons of NR because increased nitrile production could only meet 1/3rd of the new demand.

The new National Biotechnology and Biomanufacturing Initiative, launched by an Executive order of President Biden, “… *will ensure we can make in the United States all that we invent in the United States*” and “*will create jobs at home, build stronger supply chains and lower prices for American families*.” [10,11]. The press release explained that while the U.S. may be a leader in biotechnology and synthetic biology, it does not have the ability to translate discovery into practice in-house when the U.S. has to go abroad to source raw materials “… *unless we really expand the biomanufacturing base and infrastructure here in the United States*.” Domestic NR production directly addresses this deficiency.

### 1.2 Pioneers of rubber plant discoveries

**Fig. 2:**
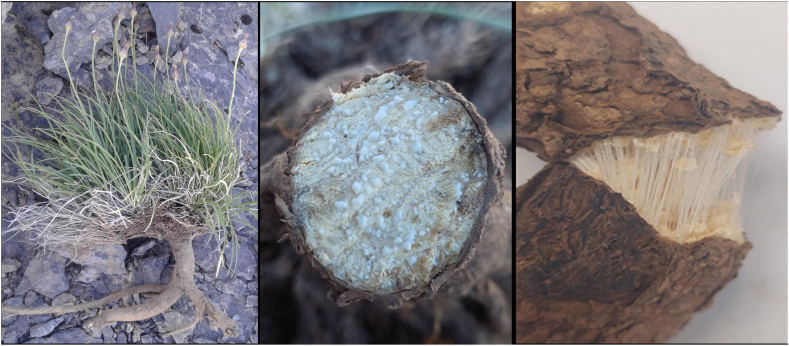
*Scorzonera tau-saghyz*, a) whole dug out plant with roots, b) latex droplets bleeding from a cut root cross-section, c) rubber threads visible as a dried root is fractured.

A paper published in **Nature** in 1945, entitled **“Russian Rubber Plants”**, described pioneering efforts to establish sources of NR independent of the tropics [12]. The search for rubber-bearing plants by the U.S.S.R. began during WWI with intentional regional expeditions. The first plant of this class, to show promise as a potential commercial source of cultivated rubber, was *tau-saghyz* (*Scorzonera tau-saghyz*), discovered in 1929 in Central Asia (Fig. 2.). The Kazakh name, “*tau-saghyz*”, translates to “mountain gum”. Native *tau-saghyz* plants with 38–40 wt% rubber content in dry roots were reported [13]. The species is hermaphrodite (has both male and female organs), is self-fertile, and is cross-pollinated by insects [13]. During the years following its discovery, more than 12 million wild plants were harvested [14]. During WWII, about 908 tons of “tau rubber” were produced for the U.S.S.R.’s defense industry [12,15].

Other Central Asian rubber-bearing species also were identified, such as *krim-saghyz* (*Taraxacum megalorrhyzon*) and *kok-saghyz* (*T*. *kok-saghyz*), or blue gum, commonly known as rubber dandelion (or erroneously called “Russian” dandelion) in some English-speaking countries. *T. kok-saghyz* and guayule (*Parthenium argentatum*), which has a rubber history dating back to Mezo-American times, have shown the most promise as rubber producers in temperate regions. Both the U.S. and the U.S.S.R. considered guayule, early on, but this crop was only developed in the U.S. because it is native to the Chihuahuan desert of Texas and Mexico, giving the U.S. easy access to species diversity in wild plants. In contrast, breeding stocks in the U.S.S.R. were based on a single guayule sample. The development of American varieties was monopolized by a private firm (the Intercontinental Rubber Company) until 1942 and this probably discouraged public breeders, before then, from guayule research. Guayule germplasm improvement, since WWII, has primarily been through selection. Hybridization is challenging because vigorous lines exhibit a range of different polyploidy (different numbers of chromosome sets) and all polyploids are either fully or facultatively apomictic [16] (where seeds are produced by asexual reproduction without fertilization). Individual wild plants containing 24 wt% (defoliated branch dry weight basis) have been found, but publicly available accessions only contain 5-7 wt%. Also, since guayule is a perennial shrub, that may live for at least 40 years, the accumulation of rubber is dependent on age. It is not yet known if guayule can be selected or modified to rapidly accumulate significantly higher concentrations of rubber in 1 or 2 years. Modern researchers have used whole plant harvests of three year old shrub or a continuous production model in which shrub is grown for two years, the tops are harvested near the ground then the regrown tops are harvested on an annual basis.

An interesting aspect of past efforts is that seeds of three rubber species (*tau-saghyz, krim-saghyz and kok-saghyz*) were obtained from the U.S.S.R. by the U.K. during WWII and small trials were carried out at the Royal Botanic Gardens, Kew, and elsewhere. *Tau-saghyz* appeared unsuited to the climate, but plants of *kok-saghyz* and *krim-saghyz* were raised successfully, and good quality rubber was extracted on a laboratory scale. However, plant yields were much lower than those obtained from Hevea plantations. Thus, the Nature paper cited earlier [12] concluded: “*From the point of view of world economy they* (these plants – sic.) *may be of comparatively little value, and it is only when self-sufficiency is desired that they become of general economic importance.*” **Well – the time for self-sufficiency may have arrived.**

### 1.3 Threats to domestic rubber supplies

Multiple past efforts to achieve U.S. natural rubber independence were unsuccessful, because they were funded mostly during supply chain crises [[3], [4], [17], [18], [19], [20], [21], [22], [23], [24], [25], [26], [27]]. The “Emergency Rubber Project” of WWII cultivated alternative NR-producing plants, with major plantings of guayule, rubber dandelion and Madagascar rubber vine [28], but the program was abandoned after Southeast Asian supply lines reopened. Guayule was planted domestically again in response to the Oil embargo in the 1970s, which caused very high petroleum (and SR) prices, but again the program was abandoned after the crisis ended. The most recent funded R&D efforts in guayule rubber (GNR) and rubber dandelion rubber (TNR) are limited in product scope (primarily tires), such as the Continental Tire-led European rubber dandelion academic/industrial program, China's 19-member rubber dandelion consortium funded by Ling Long Tire, EU-10.13039/501100011036PEARLS dandelion and guayule consortium, Drive-4-EU guayule research in France and Spain, Spain's consortium funded by Nokian Tire, and Bridgestone America's-led guayule consortium in Arizona (a follow-on from Cooper Tire's 10.13039/100000199USDA BRDI project), which included federal and academic partners. Guayule latex (GNRL) has received federal funds for a medical radiation attenuation glove. The Ohio State University (OSU) leads the interdisciplinary Program of Excellence in Natural Rubber Alternatives (PENRA) consortium and has significantly advanced rubber crop biology and rubber science. These recent scientific advances and world events, such as COVID-19, set the stage for a dramatic change of course. COVID-19 and the war in Ukraine have painfully exposed critical supply chain problems related to globalization.

This paper demonstrates that S. *tau-saghyz* roots collected in Kazakhstan contain sufficient high molecular weight NR to merit their further development as an alternative rubber crop.

## 2 Methods

### 2.1 Water-assisted mechanical extraction

The following rubber extraction method was based on an indigenous method in Kazakhstan used to make natural chewing gum. Mature *S. tau-saghyz* (STS) plants (2–5 years old) were harvested (dug out from the ground) on June 16, 2021, from wild stands in the Karatau mountains, growing on the Kumantas ridge, and from Saraba. Plants were shaken by hand to remove adhering substrate (sand and rocks). The plants were dried outdoors for 1–2 weeks until a constant dry weight was reached. The roots, 0.5–3 cm in diameter, were then cut from the plants and reweighed before rubber extraction. The roots were chopped into 5–10 cm pieces. then beaten with a hammer to break down the bark and other plant tissue. After a few hammer blows, loosened pieces of root bark and cortex were removed by hand and by slightly stretching the rubbery root piece in different directions to encourage cortical fragments to be released from the surface of the rubbery strands (Fig. 2). After no more fragments were released, each sample was rehammered and the process repeated 2–4 times to generate a partially purified sample. Each sample was then stretched and manipulated underwater to release fine cortical particles and any residual root bark. This washing process was repeated twice more. The washed sample was dried to constant weight and weighed. All the nonrubber fragments released throughout the purification process were collected, pooled, dried and weighed. The yield was then calculated based on the dry weight of the original roots.

### 2.2 Rubber purification

Approximately 40 mg of rubber from STS1 were dissolved in 20 mL THF, and the sediment and supernatant were separated. The sediment (containing non-rubber root and insoluble rubber gel) and supernatant (containing dissolved rubber) were dried and weighed.

### 2.3 SEC analysis

SEC measurements were performed using a system consisting of an Agilent 1260 infinity isocratic pump, a Wyatt Eclipse DUALTEC separation system, a Wyatt OPTILAB T-rEX interferometric refractometer, a Wyatt DAWN HELOS-II multi-angle static light scattering detector (MALS), an Agilent 1260 infinity standard autosampler, and 6 StyragelVR columns (HR6, HR5, HR4, HR3, HR1, and H0.5) thermostated at 35 °C. Tetrahydrofuran (THF), continuously distilled from CaH_2_, was used as the mobile phase at a flow rate of 1 mL/min. The dried purified rubber was redissolved in freshly distilled THF to make a 2 mg/mL solution. 1 mL of the solution was filtered through 0.45 μm PTFE syringe filters into SEC vials. In every case, 100 μL was injected into the SEC and the results were analyzed using ASTRA 7 software (Wyatt Technology). Absolute molecular weights were obtained using Polystyrene: dn/dc = 0.185 mL/g; polyisoprene: dn/dc = 0.130 mL/g.

## 3 Results and discussion

### 3.1 Rubber extraction, purification and quantification

Rubber yield data for three roots from two mature *S. tau-saghyz* (STS) plants (2–5 years old) are given in Table 1 (STS1.1 indicates plant 1, root one, STS1.2 indicates plant 1, root 2 and so on). Water-assisted mechanical extraction of dried roots yielded 27–32 wt% solid material (see Methods, Table 1).

**Table 1:** Rubber yields from water-assisted mechanical extraction.

Root section	Sectionweight (g)	Rubber^a^ (g)	Rubber^a^ wt%
STS1.1	2.90	0.864	29.8
STS1.2	2.22	0.703	31.6
STS1.3	2.40	0.746	31.1
Average			30.8
STS2.1	5.66	1.525	26.9
STS2.2	6.66	1.559	23.4
STS2.3	5.28	1.690	32.0
Average			27.4

a Rubber and leftover debris.

After the addition of tetrahydrofuran (THF) two layers formed: a supernatant and a gel-like layer at the bottom. After separation of the two phases and evaporation of the THF, the mass of solids recovered from the two phases (m_sup_ and m_sed_, supernatant and bottom layer, respectively) agreed with the starting mass within experimental error (Table 2). The insoluble fraction contained lignocellulosic debris and swollen gel, and the rest was soluble rubber.

**Table 2:** Purification of raw rubber samples after water-assisted mechanical extraction.

Sample	Rubber^a^ (m_r_) (g)	Dried supernatant (m_sup_) (g)	Dried bottom layer (m_sed_) (g)	% insoluble	rubber (g)	% rubber/root section
1	0.0401	0.0246	0.0197	49.13	0.4482	15.45
2	0.0405	0.0283	0.0191	47.16	3714	16.73
3	0.0406	0.0245	0.0192	47.29	3932	16.38
Average						16.19

a rubber and debris from water-assisted mechanical extraction.

**Fig. 3:**
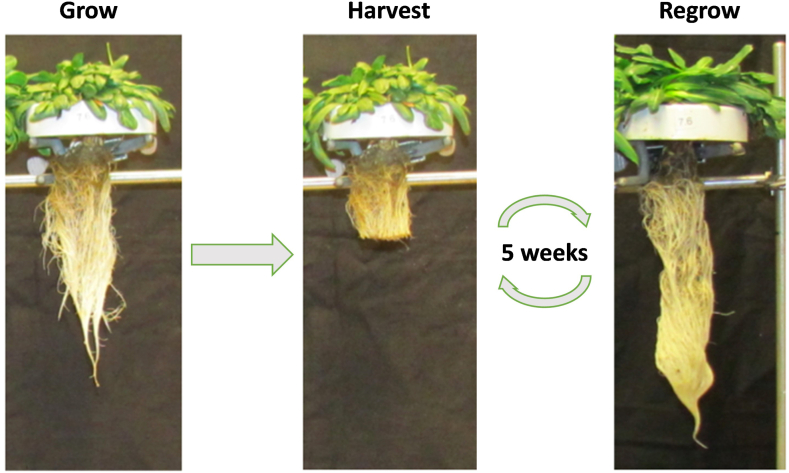
Hydroponic *Taraxacum kok-saghyz*. Roots rapidly regrow after initial harvest and contain the same concentration of latex/rubber.

Overall, 16.2 wt % THF-soluble rubber was recovered from the dried root. In comparison, rubber content in most rubber dandelions is ∼4–8 % although 22.8 wt % has been reported [29,30]. Also, the large size of *S. tau-saghyz* roots may lead to a more robust rubber production system than the smaller rubber dandelion. In addition, the roots can regrow, similarly to rubber dandelions. A hydroponically grown rubber dandelion, in a system developed by Cornish (Fig. 3), showed that after cutting the roots and recovering latex and rubber, they regrow in 5 weeks, allowing multiple harvests of the same plants. Although this report is on soil-grown STS plants, it may be possible to grow them hydroponically and this will be explored in future research.

It is also important to note that while latex and rubber are located in laticifers (“drain tubes”) in Hevea and rubber dandelions, they are inside bark parenchyma cells in guayule.

### 3.2 Macromolecular characteristics

**Fig. 4:**
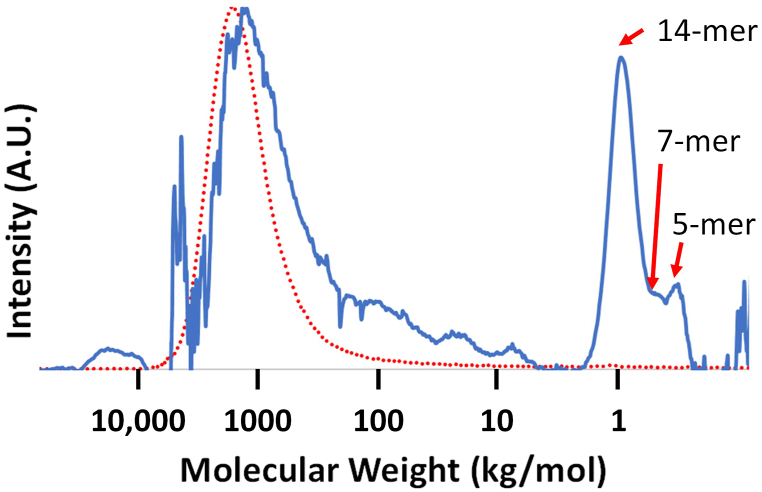
SEC traces of a representative sample. dRI (blue) concentration, LS (red) traces vs molecular weight. (For interpretation of the references to colour in this figure legend, the reader is referred to the Web version of this article.)

Purified *S. tau-saghyz* rubber samples were analyzed by high-resolution multidetector SEC that measures absolute molecular weights and other parameters from the different detectors (Fig. 4). The blue trace is the differential Refractive Index signal (dRI) that measures the concentration of the sample. The red line is from the Multiangle Laser Light Scattering (MALS) detector which measures absolute molecular weight. The dRI trace shows two distinct regions: the left is high molecular weight (long chain) polymers, whereas the right region is related to oligomers (short chains). The areas under the dRI signals are proportional to concentration, showing that the oligomer fractions are substantial (∼40 wt%). Similar oligomer fractions were reported for Hevea and guayule rubbers [[31], [32], [33], [34]]. The MALS detector is not sensitive to low molecular weight species, so no signal is seen in that area. The average data from three samples is summarized in Table 3. The mass recovery of 70 % reflects a 30 % gel fraction which did not pass through the SEC filter.

**Table 3:** SEC data.

M_w_ (kg/mol)	M_n_ (kg/mol)	Dispersity (Ɖ) (M_w_/M_n_)	Polymer	Oligomer	Gel
			Mass recovery (%)		(%)
1223.9	563.1	2.20	50.5	20.0	29.5

According to the SEC data, the rubber from *S. tau-saghyz* has a high molecular weight (above a million g/mol) with ∼20 wt% oligomer fraction and ∼30 wt% gel. Hevea rubber, obtained by tapping the aqueous latex from laticifers and then coagulating it for dry rubber applications, also has an M_w_ over a million g/mol with an oligomer fraction of 30–50 % and a gel fraction of also about 30 wt% [31,35,36]. Thus, *S. tau-saghyz* rubber is very similar to Hevea rubber and should be able to produce a full range of high-performance rubber products.

### 3.3 Need for additional research

Cornish and Puskas (OSU) have pioneered academic research, together with the USDA, and many other international groups have made significant contributions throughout recent years [2,20,[37], [38], [39], [40], [41], [42]]. Despite a nearly 4000-year history and the genetic tools of modern biology, we still do not fully understand how NR is made by plants. The exact structure of Hevea rubber, the commercially used NR, is still unknown, and no one has been able to isolate and fully characterize the enzyme complex catalyzing NR biosynthesis [37,43,44]. New research is essential to unravel the remaining mysteries of natural rubber biosynthesis. Renewed U.S. research could catalyze the production of up to 30 million acres of domestic rubber crops with a societal impact of capturing ∼200 Mt (∼5 %) of U.S. CO_2_ emissions and converting them into rubber by the new crops, and ∼3 million jobs tied to U.S. soil. Demand has been growing exponentially (Fig. 1) [6,7]**,** and has recovered from the sharp drop seen in 2020 [9]. However, demand has been always close to supply. For example, in 2022 production was 14.47 million tons and demand was14.31 million tons and is projected to exceed supply by 40,000 tons in 2023 (production 14.693 million tons and demand 14.738 million tons) [[45], [46], [47]]. Total dependence on foreign sources threatens U.S. rubber security, as clearly experienced during the recent human disease outbreaks and supply disruptions [8,48]. During WWII, academe, government and industry worked together with federal investment equivalent to $11.1 billion in today's dollars to domestically produce and process rubber, breaking down all barriers, sharing information and resources. Can we do it again?

## 4 Conclusion

*S. tau-saghyz,* with its high concentration of ∼16–40 wt% of the root mass, high rubber molecular weight and its adaptability to temperate climates with cold, snowy winters, is a very promising renewable source for critically needed domestic rubber. More detailed investigations of this very interesting rubber-producing plant are in progress.

## CRediT authorship contribution statement

**Judit E. Puskas:** Conceptualization, Funding acquisition, Investigation, Methodology, Resources, Supervision, Writing – original draft, Writing – review & editing. **Katrina Cornish:** Conceptualization, Funding acquisition, Investigation, Resources, Supervision, Writing – original draft, Writing – review & editing. **Boguspaev Kenzhe-Karim:** Conceptualization, Formal analysis, Funding acquisition, Methodology, Resources, Supervision, Writing – original draft, Writing – review & editing. **Meirambek Mutalkhanov:** Formal analysis, Investigation, Methodology, Validation, Visualization, Writing – original draft, Writing – review & editing. **Gabor Kaszas:** Formal analysis, Investigation, Methodology, Writing – original draft, Writing – review & editing. **Kristof Molnar:** Formal analysis, Investigation, Methodology, Supervision, Writing – original draft, Writing – review & editing.

## Declaration of competing interest

The authors declare the following financial interests/personal relationships which may be considered as potential competing interests:Katrina Cornish reports financial support was provided by US Department of Agriculture. Judit E. Puskas reports financial support was provided by US Department of Agriculture. Kristof Molnar reports article publishing charges was provided by Semmelweis University. Boguspaev Kenzhe-Karim reports financial support was provided by Ministry of Science and Higher Education of the Republic of Kazakhstan.
